# Surgical Treatment for Scaphocephaly Using a Modified Melbourne Technique in Mexico: An Illustrative Case and Literature Review

**DOI:** 10.7759/cureus.73089

**Published:** 2024-11-05

**Authors:** Roy Ferrufino Mejia, Héctor A Rodríguez-Rubio, Mayra Alejandra Arce-Lozoya, Shirley Rocío Chavarría-Mejía, Flavio Hernandez-Gonzalez, Osvaldo Manuel San Martín-García, Yamile Giovanna Serrano-Pinto, Alan Ferrufino-Mejia

**Affiliations:** 1 Neurosurgery, Mexican Institute of Social Security (IMSS) XXI Century National Medical Center, Mexico City, MEX; 2 Neurosurgery, Juarez Hospital of Mexico, Mexico City, MEX; 3 Neuroanesthesiology, Mexican Institute of Social Security (IMSS) XXI Century National Medical Center, Mexico City, MEX; 4 Neurosurgery, Centro Medico Nacional "20 de Noviembre, Instituto de Seguridad y Servicios Sociales de los Trabajadores del Estado (ISSSTE), Mexico City, MEX; 5 Neuroinfectology, Clínica Especializada "San Fernando, Instituto de Seguridad y Servicios Sociales de los Trabajadores del Estado (ISSSTE), Mexico City, MEX

**Keywords:** craniosynostosis, melbourne method, pediatric neurosurgery, sagittal synostosis, scaphocephaly

## Abstract

Craniosynostosis is the premature fusion of one or more skull vault sutures, most commonly the sagittal sutures, leading to a long, narrow head shape known as scaphocephaly. Surgery is recommended to create space for brain growth to treat scaphocephaly. Delayed treatment may require more complex surgery to achieve the desired head shape. The Melbourne method of total calvarial vault reconstruction allows for significant head shape change without requiring postoperative helmet therapy. This single-stage technique involves complex skull reshaping to move the vertex successfully from front to back by reconstructing both the anterior and posterior parts of the cranium. We present the case of an eight-month-old boy diagnosed with nonsyndromic scaphocephaly, characterized by an elongated head with a prominent sagittal ridge due to secondary sagittal synostosis. Subtotal cranial vault reconstruction was performed with good postoperative results.

## Introduction

Craniosynostosis is the early fusion of skull vault sutures, and it affects about one in 2000 live births. Scaphocephaly, caused by isolated sagittal synostosis, makes up around 50% of cases and occurs at a rate of 1.5 per 10,000 live births [1]. The exact prevalence of craniosynostosis in Mexico is currently unknown. However, the National Institute of Pediatrics in Mexico City reported 157 cases of the condition in 2016 [2]. Additionally, from 2010 to 2016, Hospital "La Raza" in Mexico treated 196 cases of nonsyndromic scaphocephaly [3].

Operative intervention is typically done before 12 months of age to reduce the risk of elevated intracranial pressure and limited bone healing capacity. Treatment involves surgery, with newer techniques like extended strip craniectomy using endoscopic methods being more common [4]. Dynamic methods, such as the Pi procedure, are used in younger patients, while older patients may require more extensive reconstructive procedures due to reduced bone healing capacity [5].

The "Melbourne technique" is a radical method for addressing scaphocephalic deformity. It involves frontal and occipital reconstruction and the unique step of rotating and replacing a strip of bone to correct the low-lying posterior vertex and increase intracranial volume. Total cranial vault remodeling using the Melbourne technique can make a more regular head shape without reducing calvarial volume. It addresses frontal bossing, acute nasofrontal angle, and anteriorly displaced vertex, compensating for the volume lost by the anterior-posterior squeeze [6].

Three-dimensional (3D) are used to plan complex procedures and generate intraoperative guides to predict operative morphologic outcomes accurately. This helps us objectively plan the desired head shape before surgery [7,8]. There is limited published evidence on using the Melbourne technique for craniosynostosis in Mexico. This report outlines our surgical experience with a modified Melbourne technique for total calvarial vault remodeling in patients with late-presenting isolated sagittal synostosis.

## Case presentation

**Figure 1:**
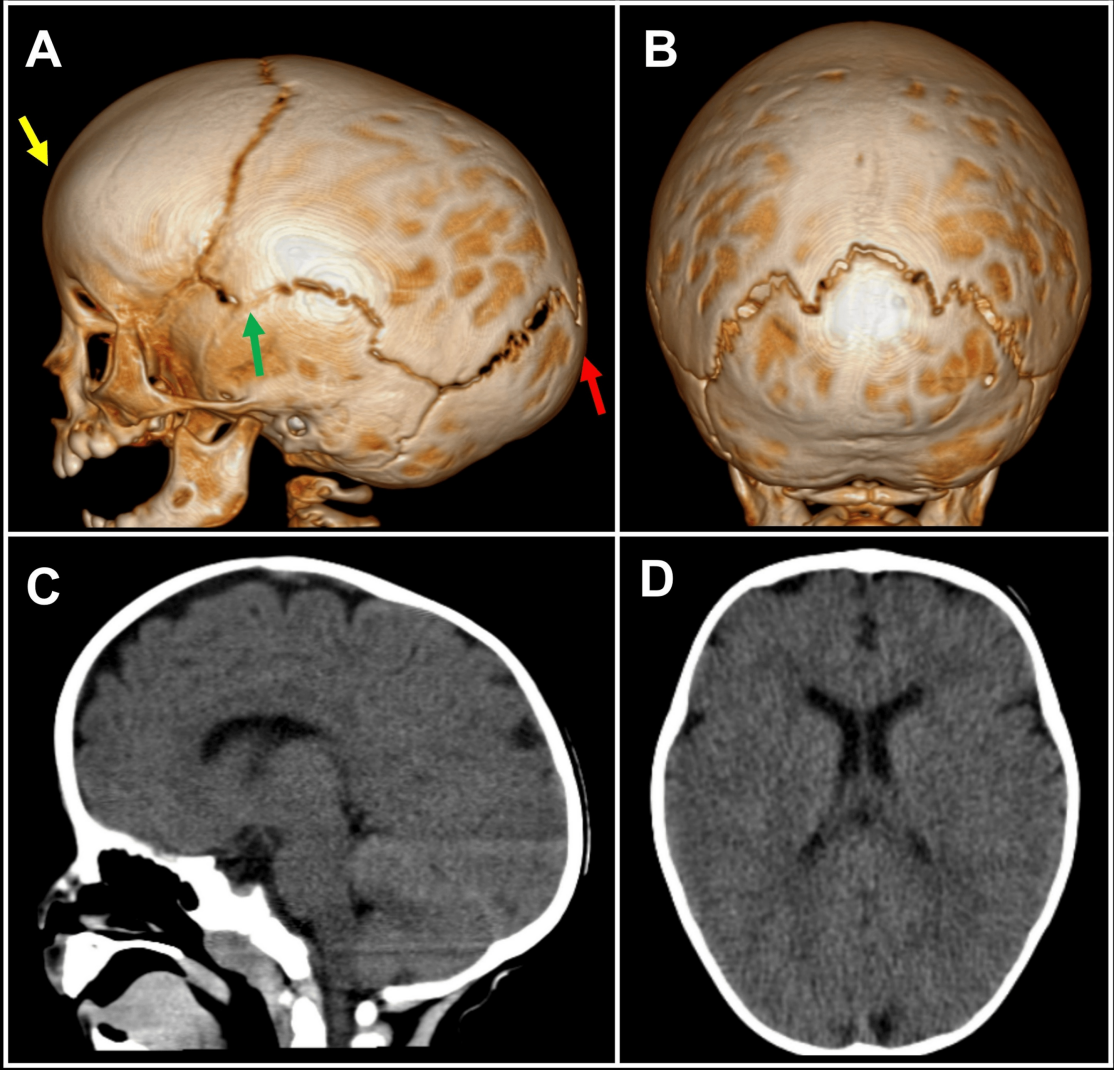
Preoperative CT and 3D reconstruction Complex nature of the nonsyndromic scaphocephalic deformity, with frontal bossing (yellow arrow), temporal in-drawing “pinching,” an anteriorly placed vertex (green arrow), an occipital “bullet” deformity, and also craniolacunia (red arrow). CT: computed tomography, 3D: three dimensional

An eight-month-old boy presented with nonsyndromic scaphocephaly. His history includes being born at 36 weeks gestation as a result of in vitro fertilization and delivered vaginally. He has no history of vomiting, headaches, or blurred vision. No formal measurements of intracranial pressures were taken before the surgery. On examination, an elongated head with a prominent sagittal ridge was observed, which was determined to be due to secondary sagittal synostosis and decreased biparietal diameter. A 3D CT revealed a long, narrow head shape with premature sagittal suture closure (Figure 1).

**Figure 2:**
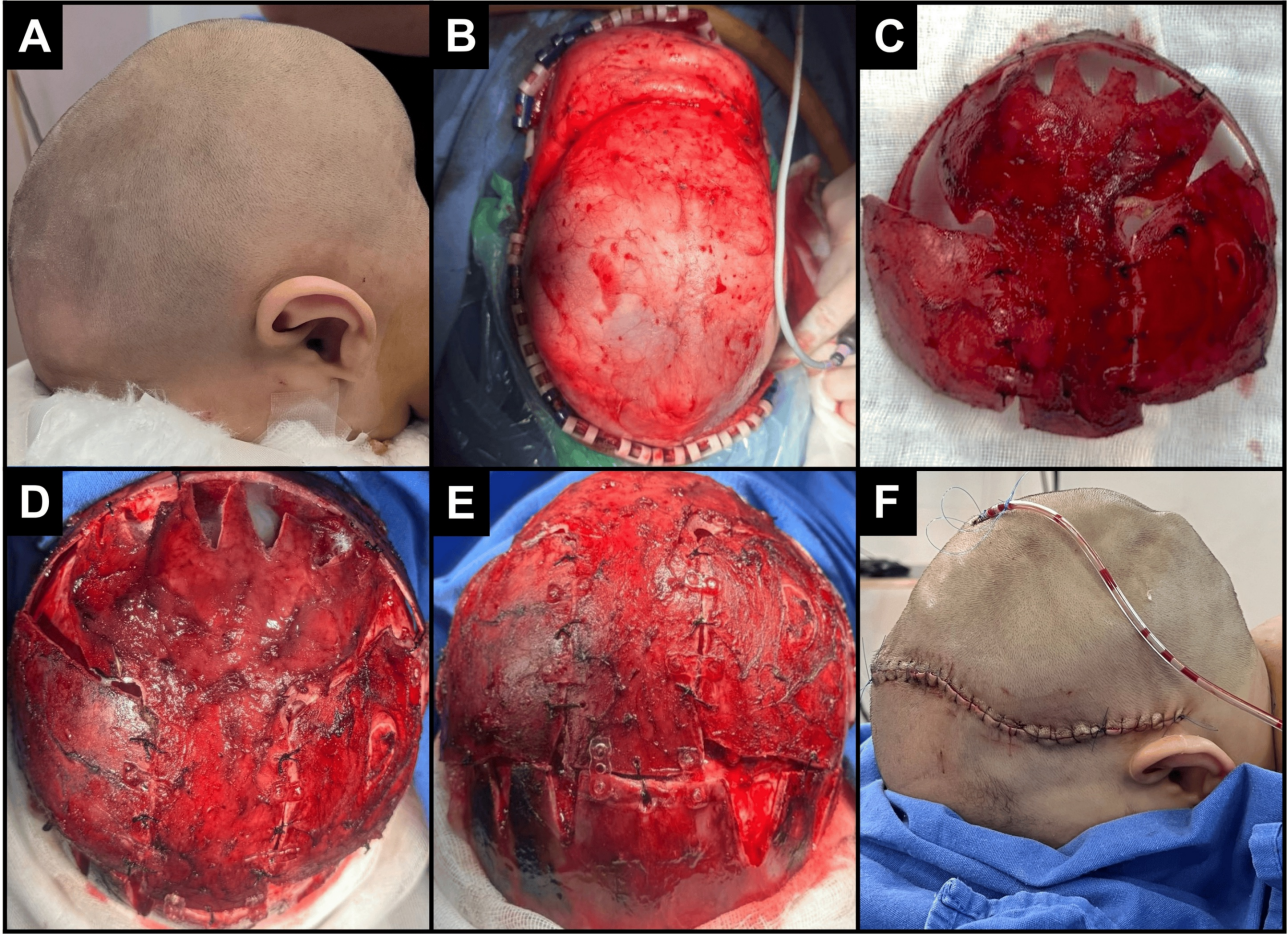
Surgical aspects (A) Cranium with anterior and posterior lengthening in a modified prone position with slight head extension. (B) The Melbourne procedure: view of the standard biparietal incision. (C) Dissection and removal of bone flap. (D) View of dissected area. (E) Views after osteotomies and remodeling. With the new position of the cranial strip, it was divided and repositioned to widen and elevate the occipital region. (F) Surgical closure.

During the procedure, the patient was positioned on his stomach (prone) with a slightly extended head and a pad under the thoracic region for support, allowing for exposure from the front (anterior) to the top (bregma) and back (posterior) to the bottom (lambda) of the head (Figure 2A). An incision was made between bregma and lambda, just above the ear, and was treated with epinephrine 1:400,000 to minimize bleeding during the procedure. The craniectomy was marked with bregma as the front limit and lambda as the back limit, with a 3-4 cm cut on each side, while special care was taken around the sagittal sinus (Figure 2B). The bone flap was then removed in one piece (Figure 2C). After performing relaxation cuts at the temporoparietal level to promote remodeling of the biparietal diameter, which was found to be reduced in cases of sagittal suture fusions, we applied hemostatic agents (Figure 2D). The galea was closed with 3-0 Vicryl and the skin with 4-0 nylon or metallic staples (Figure 2E).

**Figure 3:**
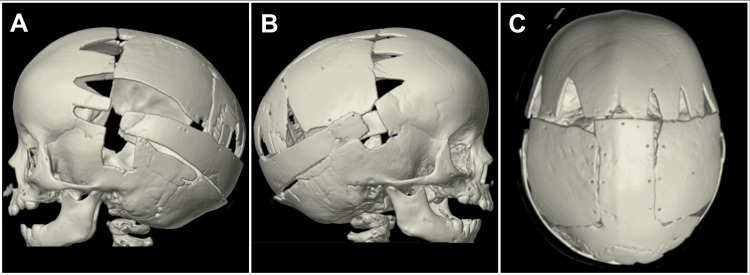
Post-surgical CT 3D reconstruction Demonstrating the modified Melbourne procedure. Our modifications include using a more formal fronto-orbital remodeling technique rather than the simple barrel-staving of the "F" flap. We also use morsellized bone paste "salami" to fill all bone defects during the surgery. CT: computed tomography, 3D: three dimensional

Additionally, a 1/8 subgaleal drain was placed, being careful not to position it over the sagittal sinus to avoid the risk of injury when removing the drain (Figure 2F). No postoperative cranial defects were identified during the follow-up (Figure 3).

## Discussion

Patients diagnosed and treated early for scaphocephaly often face challenges due to limited awareness and confusion with other cranial abnormalities. Socioeconomic factors may further delay treatment. Late recognition and surgical intervention can lead to complications such as papilledema, increased headaches, and bony ingrowth failure [9]. Studies like Paige et al. [10] show that ossification rates decline with age. In cranial vault reconstruction, surgery performed before nine months enhances postoperative ossification. One study suggests optimal neurological development occurs with surgery before six months, while another recommends it be done before 12 months [11].

Minimizing the number of surgical stages is especially important for older, more socially aware patients, as children typically become self-conscious around the age of 2. In a 30-year institutional study, Utria et al. [11] identified an ideal timeframe for surgery to be between nine and 12 months. Non-surgical molding helmet orthoses are gaining popularity, but surgery is still the primary treatment [12]. Spring-assisted cranioplasty is preferred for small infants with nonsyndromic scaphocephaly at Great Ormond Street Hospital's Craniofacial Unit due to quicker surgeries and shorter hospital stays [13]. However, this method is unsuitable for older children who need different approaches.

The surgical management of scaphocephaly requires a trained multidisciplinary team for pediatric patients. Effective preoperative, intraoperative, and postoperative care is vital to reduce complications. The recommended position for early treatment is ventral decubitus with cephalic extension, allowing for occipital osteotomies if necessary [14]. To correct severe scaphocephaly, the focus is on addressing biparietal narrowing, frontal bossing, and the displaced vertex while allowing for brain expansion. Current methods reduce the anteroposterior length and increase biparietal width but do not adequately remodel the frontal bone or occiput. The total cranial vault approach has several benefits in cases of late scaphocephaly. It allows for custom tailoring of the skull to minimize defects and control the vertex position by repositioning bony segments, effectively addressing deformities [15]. The Melbourne method uses diagrams and relies on the surgeon's freehand markings for cutting and repositioning. It aims to elevate the vertical occipital fasciculus area to adjust the vertex location, promoting brain expansion while reducing the risk of increased intracranial pressure. However, this approach has limitations in predictability and outcome accuracy. Challenges include determining ideal bone segment sizes and osteotomy cutting sites and accurately predicting postoperative dimensions and vertex height [16].

Sharma et al. [7] modified the Melbourne technique for cranial vault reconstruction to include a two-stage surgery under the same anesthesia, changing from supine to prone. They reported no issues despite potential risks like anesthetic complications and contamination. The Melbourne technique treats both the front and back of the head simultaneously. Instead of performing straightforward surgeries, this method focuses on removing and lifting the entire back of the head. There are concerns about sagittal synostosis surgeries, especially regarding their potential effects on skull growth and intracranial volume [17].

When performing a single-stage total vault reconstruction, it's important to note that the procedure can be lengthy and may lead to significant blood loss, often requiring transfusion. Although transfusions carry some risks, complications are rare. We typically use arterial lines for monitoring. Safety in craniosynostosis surgeries has improved over the years, and older patients generally experience less blood loss proportionately [18]. This technique effectively addresses severe scaphocephaly in young patients. Our modified method reduces bone graft movement, leading to shorter surgeries and less need for transfusions. While early results are promising, further validation in a larger group and follow-up is needed.

## Conclusions

A modified version of the Melbourne method for total calvarial vault reconstruction aims to address the various phenotypic features of severe scaphocephaly associated with isolated sagittal synostosis while maintaining structural integrity across the calvaria. Our initial experiences indicate that this procedure produces favorable results that outweigh the minimal additional risks involved. However, achieving good results during surgery does not necessarily guarantee an overall successful outcome. More surgeries and medium- to long-term follow-ups are necessary for a thorough evaluation.
